# Thermal Behavior of Graphene Oxide Deposited on 3D-Printed Polylactic Acid for Photothermal Therapy: An Experimental–Numerical Analysis

**DOI:** 10.3390/jfb14020080

**Published:** 2023-01-31

**Authors:** Jesús Vence, Christian Gil, Laura González-Rodríguez, Miriam López-Álvarez

**Affiliations:** CINTECX, Universidade de Vigo, Campus Universitario Lagoas-Marcosende, 36310 Vigo, Spain

**Keywords:** graphene, phototherapy, simulation, laser, PLA

## Abstract

The present work evaluates the thermal behavior of graphene oxide (GO) when deposited on 3D-printed polylactic acid (PLA), in order to develop a medical device for photothermal therapy applications. An experimental–numerical analysis was performed to assess the photothermal conversion capacity, based on the power emitted by a NIR (785 nm) laser, and the subsequent temperature distribution on the GO-PLA material. The influence of the deposited mass of GO and the PLA thickness was studied through 40 different scenarios. The results estimated a value of photothermal conversion efficiency of up to 32.6%, achieved for the lower laser power density that was tested (0.335 mW/mm²), and a high mass value of deposited GO (1.024 × 10^−3^ mg/mm²). In fact, an optimal mass of GO in the range of 1.024–2.048 × 10^−3^ mg/mm^2^ is proposed, in terms of absorption capacity, since a higher mass of GO would not increase the conversion efficiency. Moreover, the study allowed for an estimation of the thermal conductivity of this specific biomaterial (0.064 W/m·K), and proved that a proper combination of GO mass, PLA thickness, and laser power can induce ablative (>60 °C, in a concentrated area), moderate (50 °C), and mild (43 °C) hyperthermia on the bottom face of the biomaterial.

## 1. Introduction

Currently, the diagnosis and treatment of drug-resistant bacterial infections represent one of the greatest challenges in the biomedical field, leading to around 700,000 deaths worldwide each year [1,2]. Consequently, conscientious research to provide accurate diagnoses and discover alternative treatments is required. Hyperthermia has lately become one of the most intensively explored strategies, given the proven efficacy of induced mild (≤43 °C [3]) and moderate (≤50 °C [4]) warming-up against drug-resistant strains [5,6,7]. In relation to this finding, it has been proven that an abrupt increase in temperature can destroy the bacteria via various thermal effects, such as the breakdown of the cell membrane, which leads to the leakage of cellular content or the denaturation of the proteins/enzymes that mediate most of the physiological activities in microbes [8,9,10].

One strategy to locally induce hyperthermia is photothermal therapy (PTT), using proper photothermal conversion agents (PTCAs) in contact with the damaged tissue. More concisely, PTT mediated by near-infrared radiation (NIR) is especially interesting, given that a low-energy NIR light presents a relatively high tissue penetration depth, compared to visible and UV light, and also avoids skin burns [5,11,12]. Furthermore, it requires low laser power and short time interactions [7]. Apart from these advantages, other positive issues can be highlighted: spatiotemporal addressability, minimal invasiveness, relative clinical safety, and non-drug resistance in comparison to other antibacterial strategies, such as chemotherapy, photocatalytic therapy, antibacterial peptides, and photodynamic therapy [13,14]. Moreover, the biological window of NIR I (650–950 nm) demands less expensive equipment (laser and detector) in relation to the window of NIR II (1000–1700 nm) [15]. 

The literature shows that previous research has been carried out to develop PTCAs that can absorb the NIR light in the desired window more efficiently, to treat tissues in poorly accessible regions [7]. Different approaches have been proposed, such as semiconducting polymer organic nanoparticles [13,16], noble metal materials, transition metal oxides, and carbon-based materials. Most of these agents employ the absorption of remote radiated NIR light to convert it into heat and locally trigger the death of cancer cells [8,17]. However, these PTCAs are also required to ensure minimal light absorption by the surrounding tissues and minimal retention in other healthy organs (especially in the case of nanoagents) and must avoid the fluorescence interference of biological tissues [15]. The low targeting efficiency caused by the nonspecific distribution of these phototherapeutic agents is also important to address, not only to increase bioavailability and efficacy but also to avoid side effects [13].

Among the many evaluated carbon-based PTCAs, graphene oxide (GO) is considered a very interesting option for the treatment of drug-resistant bacterial infections by NIR I-triggered photothermal therapy [7]. The advantages are not only the high capacity of NIR light absorption and good thermal stability but also its high drug-loading capacity, which makes GO a good carrier for a combined treatment of both photothermal therapy and chemotherapy [14]. Regarding the photothermal conversion efficiency of GO, Savchuk et al. [18] stated an efficiency of 58 ± 5% when irradiating GO with a wavelength of 808 nm and a laser power of 200 mW. This value is higher than the one reported for Au nanostructures and several semiconductor materials, polymer nanostructures, or nanoparticles with ferromagnetic properties for the same wavelength. Moreover, in comparison to other carbon-based PTCAs, GO offers better water dispersibility than both graphene and reduced graphene oxide (rGO), resulting in low cytotoxicity [4]. In relation to this latter quality, recent publications [1,19,20] have proven GO biocompatibility at certain concentrations.

Furthermore, the functionalization of GO and its combination with other biomaterials is being widely investigated. Among all the possibilities, certain polymers offer interesting properties, such as biodegradability, biocompatibility, and good processability. Some of the most investigated polymers are collagen, alginate, chitosan, polyvinyl alcohol, poly(ϵ-caprolactone), and polylactic acid (PLA), among others [21]. In the particular case of PLA, interest in this biocompatible, biodegradable, and bioabsorbable polymer has risen dramatically with the emergence in the biomedical field of fused deposition modeling (FDM), one of the most common 3D printing techniques [22]. In this regard, PLA presents thermoplastic properties and a low glass transition temperature (55–65 °C), which means that it is deformable under high temperatures (190–220 °C). Moreover, it can be heated to its melting point, cooled, and then reheated again without significant degradation [22,23]. Its use in FDM technology enables the rapid manufacture of customized devices for biomedical applications. 

The current work presents an evaluation of the thermal behavior of a GO-PLA biomaterial for NIR photothermal therapy, using 785 nm laser radiation. An experimental–numerical analysis was performed to evaluate the photothermal conversion ability of GO, when incorporated on one surface of a 3D-printed PLA disc, to induce mild (43 °C) or moderate (50 °C) hyperthermia on the opposite surface. The relationship between the power of the laser radiation and the temperature distribution across the probe was experimentally analyzed, along with the influence of the deposited mass of GO and the thickness of the probe. To extrapolate the thermal behavior of the GO-PLA biomaterial to other experimental conditions or other geometries, a numerical analysis was carried out, providing the unknown thermal conductivity of the GO-PLA biomaterial, the pattern of distribution of the temperatures, and the photothermal conversion efficiency. The present development is conceived in terms of its application as a 3D-printed PLA splint for the treatment of infections in the oral cavity, it is thus externally coated with GO on the specific areas of interest. The GO coating will be remotely irradiated with the NIR laser, inducing hyperthermia on the internal face of the PLA, which will be in direct contact with the infected gingival tissue.

## 2. Materials and Methods

### 2.1. Raw Materials

An aqueous suspension of sheets of GO (0.5 mg/mL) with an elemental content of C (49–56%), H (0–1%), N (0–1%), S (2–4%) and O (41–50%) in a 95% aqueous solution was acquired from Graphenea (Donostia, Spain).

Pellets of natural polylactic acid (PLA) composed of PLA Luminy® LX175 were acquired from Filament2print (Spain). The pellets have a higher melting point than standard PLA, an increased rate of crystallization, and a stereochemical purity of ≥99% for the L-isomer, using the Total Corbion PLA method. The main properties are summarized in Table 1.

### 2.2. GO-PLA Manufacture: 3D Printing and Drop-Coating Procedures

The PLA pellets were first subjected to the 3D printing process in a Tumaker Voladora NX Pellet 3D printer (Tumaker, Irún, Spain), obtaining a set of 3D-printed PLA discs with a diameter of 11.15 mm, ranging from 1.10 to 5.60 mm in thickness (height). This 3D-FDM (fused deposition modeling) printer is fitted with a diameter nozzle of 0.80 mm. An infill pattern with the maximum allowed density, based on perpendicularly alternating processing lines from one layer to the overlapped one, was chosen to minimize pores inside the internal structure of the probes. The main 3D printer parameters are summarized in Table 2. 

**Figure 1:**
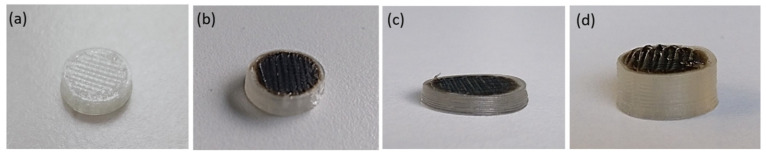
The 3D-printed PLA discs, which are 2.85 mm thick, are drop-coated with 0.00 mg of GO (**a**), with 0.10 mg of GO (**b**); 3D-printed PLA discs of 2.20 mm thick (**c**), and 5.60 mm thick (**d**), are drop-coated with 0.10 mg of GO.

Once obtained, the upper surface of the 3D-printed PLA discs was drop-coated with the GO sheets. The drop-coating technique, in comparison to others, such as the use of a combined filament in 3D printing, allows for a better knowledge of the morphology of the probe because the generation of heat is precisely located on the upper surface. In addition, this precision is a very interesting option for external medical devices because it allows for the local application of the GO to the small region in which the hyperthermia is to be generated, giving better control over the affected region, avoiding the contact of the GO with any living tissue, and minimizing the effect of the humidity of, in this case, the oral cavity. To meet this proposed application, a set (SET1) of 3D-printed PLA discs with a thickness of 2.85 mm and a deposited mass of GO, ranging from 0.01 mg to 0.20 mg, was first obtained. Then, 200 µL of the original GO aqueous suspension (0.5 mg/mL) and 200 µL from different diluted suspensions in mQ water (0.25, 0.10, and 0.05 mg/mL) were drop-coated onto the upper surface of the PLA discs. The volume of 200 µL was selected to ensure the entire coating of the PLA disc surface (diameter = 11.15 mm). These GO-PLA probes were finally left to dry in a laboratory oven at 30 °C for 24 h. Another set of samples (SET2) was subsequently obtained using 3D-printed PLA discs with the same diameter of 11.15 mm, different thicknesses ranging from 1.10 mm to 5.60 mm, and a fixed deposited mass of GO of 0.10 mg. Table 3 summarizes the two sets of GO-PLA probes; images of these GO-PLA probes are shown in Figure 1.

**Figure 2:**
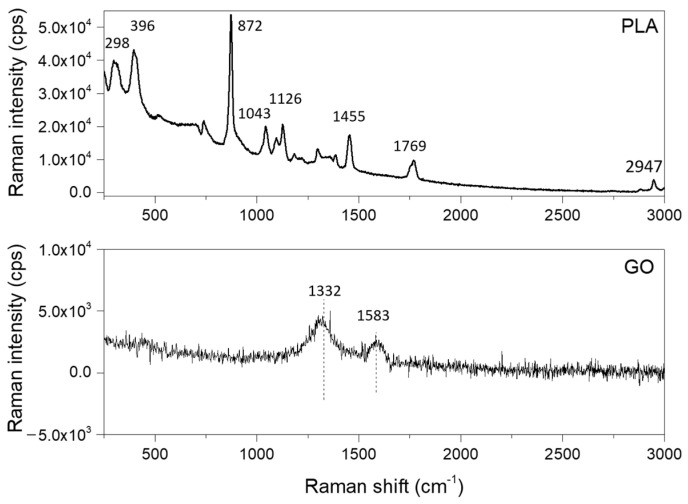
Fourier Transform (FT)Raman spectra in both faces of the GO-PLA discs.

The GO-PLA probes were characterized by Fourier Transform (FT) Raman spectroscopy to identify the main molecular vibrations and, subsequently, assign the corresponding chemical bonds. For this test, a B&WTEK i-Raman-785S instrument equipped with a BAC 100 probe (785 nm) through a 10× magnification objective was used at a resolution of 2 cm^−1^ to take 32 scans with incident laser radiation, maintained to generate 100 mW. The spectra obtained from both faces of the GO-PLA probes are shown in Figure 2. The FT-Raman spectrum obtained for the face with the GO coating revealed the typical G-band centered at 1583 cm^−1^ and a D-band centered at 1332 cm^−1^. The associated positions agree with previous works on the identification of nanosheets from GO [24]. The FT-Raman spectra for the bottom face of the 3D-printed PLA revealed the expected intense and sharp band at 872 cm^−1^, attributed to C-COO stretching, and others at 298 cm^−1^ and 396 cm^−1^, attributed, respectively, to the bending of C-O-C and C-CO; 1043 cm^−1^ was attributed to skeletal stretching in C-CH_3_; 1126 cm^−1^ was attributed to asymmetric rocking CH_3_; 1455 cm^−1^ was attributed to the symmetric bending of CH_3_; 1769 cm^−1^ was attributed to C=O asymmetric stretching; and, finally, 2947 cm^−1^ was attributed to CH_3_ symmetric stretching. In the same way, the positions for the different peaks were in line with previous works with 3D-printed PLA [25].

### 2.3. NIR Laser Source and Thermographic Cameras: Experimental Setup

**Figure 3:**
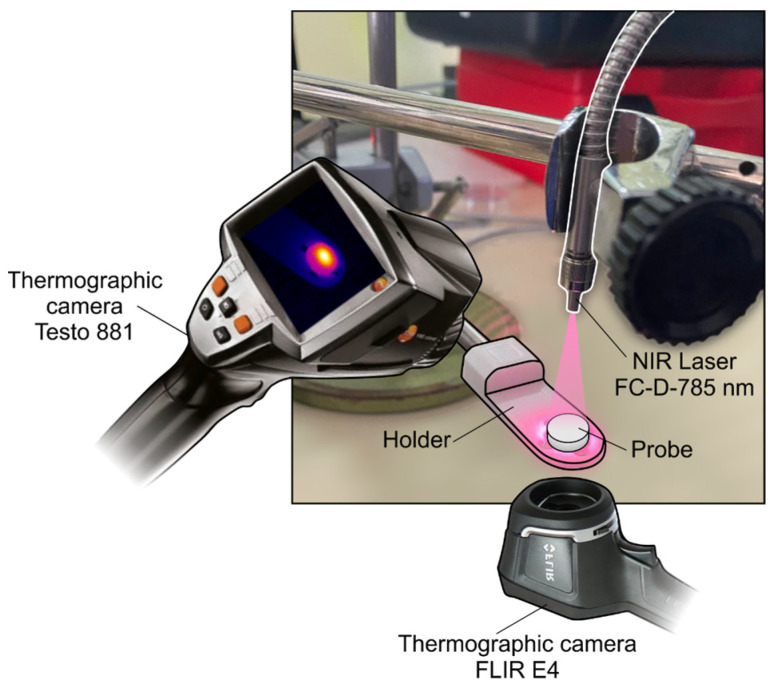
Descriptive graphic of the experimental setup using a diode NIR laser and two thermographic cameras.

The two sets of GO-PLA probes were subjected to NIR laser radiation using the experimental setup shown in Figure 3. The main objective was to evaluate the relationship between the laser radiation power and the temperature distribution from the upper surface of the probe (where the GO was deposited) to its bottom surface. Moreover, the influence on the photothermic effect of the variations in the deposited mass of GO and in the thickness of the PLA disc was also evaluated. The setup consisted of: a diode laser source FC-D (fiber coupled diode), emitting radiation of 785 nm in wavelength, with power in the range of 1–450 mW; a tube of optical fiber to guide the laser beam; a thermographic camera, Testo 881, capturing the upper surface of the probe; a thermographic camera, FLIR E4, positioned beneath the probe to capture the bottom surface. The GO-PLA probe was placed on a pierced plastic holder and received the laser radiation at its so-called “upper surface”, in the normal direction. The distance from the optical fiber to the probe was calibrated so that the beam affected it up to the boundary limits of the upper surface of the probe, meaning that all the radiation was captured by the disc. In this case, the distance was set to 32 mm from the tip of the optical fiber to the upper surface of the probe.

**Figure 4:**
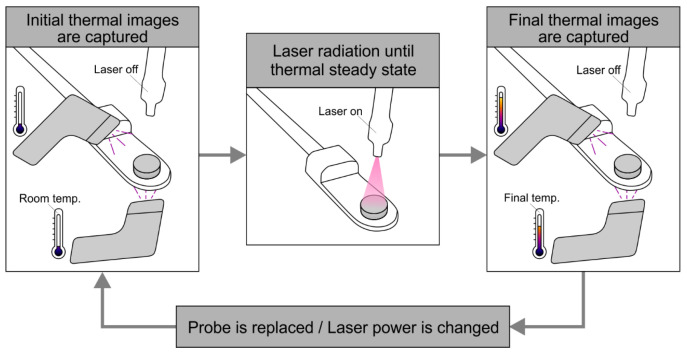
Flowchart of the experimental procedure.

**Figure 5:**
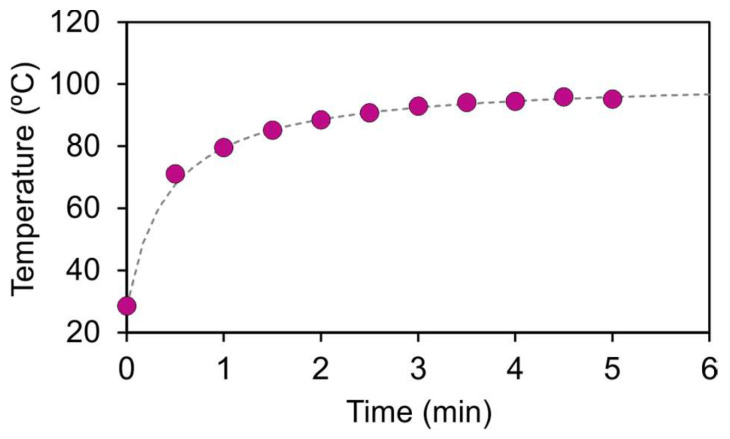
Temporal evolution of the average temperature on the upper surface of the probe.

The process for obtaining the experimental measurements is shown in Figure 4 and can be described as follows:

1. The probe was placed on the holder and both thermographic cameras captured thermal images of the initial temperature.
2. The laser was switched on and the power of the laser was set to 32.73 mW. The probe was heated until a stationary state was reached. The evolution of the temperature was captured periodically with both cameras. As shown in Figure 5 for a representative case, the stabilization time was approximately 5 min.
3. The laser was switched off and the probe was cooled down until it reached the initial temperature.
4. The process was repeated for 93.55, 194.62, and 421.67 mW of laser power, and then repeated for all the probes of SET1 and SET2.

All the thermographic images were then treated with the corresponding software (IR Soft (by TestoSE & Co. KGaA, Lenzkirch, Germany) and FLIR Tools (by Teledyne FLIR LLC, Wilsonville, OR, USA)) to extract and evaluate the profile of the temperature on both surfaces.

### 2.4. Numerical Analysis Methodology

In order to allow the extrapolation of the thermal behavior of the GO-PLA biomaterial, extracted from the experimental analysis, to other conditions or other geometries, a numerical analysis was designed and carried out. The unknown thermal conductivity of this GO-PLA biomaterial was first determined, as the patterning derived from the 3D printing process and the incorporated GO coating would modify the standard recorded conductivity of PLA.

**Figure 6:**
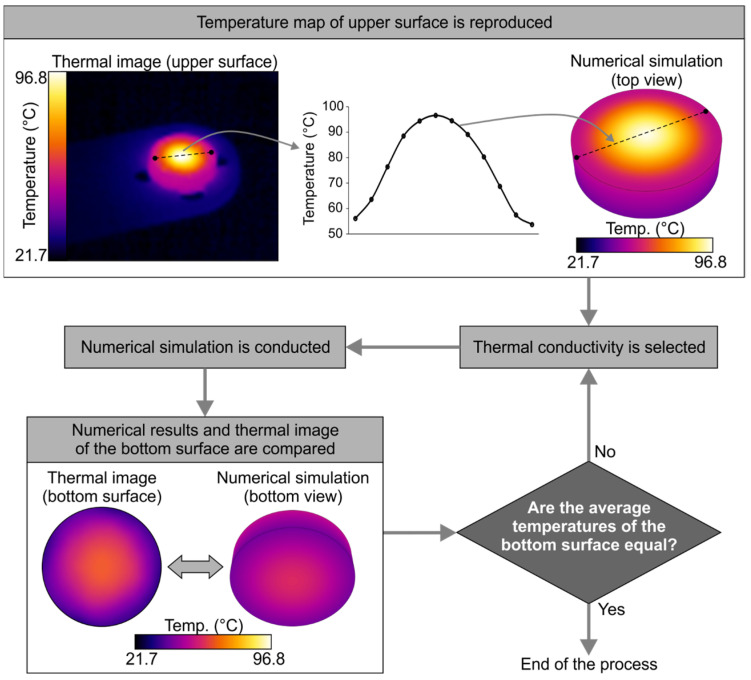
Description of the numerical process to ascertain the thermal conductivity of the probe based on the experimental results.

In line with previous studies [26,27] to determine the specific thermal conductivity of the probes, a series of steady-state numerical simulations, fed with the experimental data obtained from the thermal images, was carried out using the ANSYS Workbench (by ANSYS Inc., Canonsburg, PA, USA) software. The process is shown in Figure 6 and can be described as follows:

1. The profile of the temperature on the upper surface of the probe was extracted from the thermographic image of the stationary setup, fitted using the optimal polynomial function, and set as a boundary condition in the simulation. In this manner, the heat generation due to laser radiation was reproduced. This method was developed as an alternative to the use of Gaussian distributions [27].
2. Natural convection was applied to the probe walls, using h = 10 W/m^2^·K and T = 20 °C, and the thermal conductivity of the material was set to an initial value.
3. The simulation was conducted until it reached convergence. Then, the numerical contours of the bottom surface were extracted and compared with the corresponding thermal image. If the average temperature of the bottom surface obtained using both methods was equal, the process was finished. If not, the thermal conductivity had to be modified, and the simulation was repeated until agreement was obtained.
4. The process was performed for all 40 different scenarios that were experimentally measured.

## 3. Results & Discussion

### 3.1. Thermal Characterization of the Biomaterial

**Figure 7:**
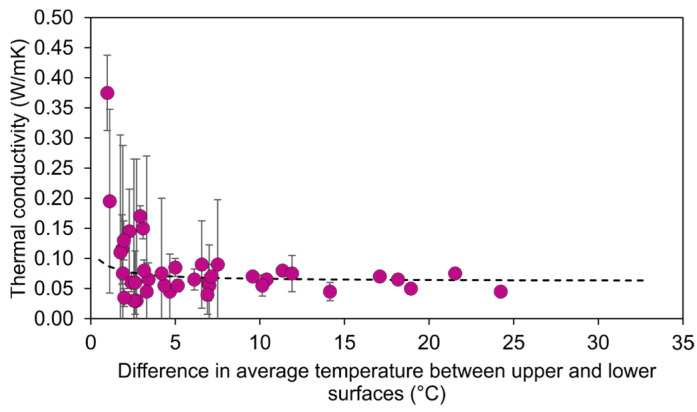
Graph of the obtained value of thermal conductivity vs. the difference in the average temperature between the upper and lower surfaces of the probe for all the experimental points.

As mentioned in Section 2.2, determination of the thermal conductivity of the manufactured material (PLA + GO) provides a crucial parameter for the extrapolation of the obtained results to other conditions and geometries. In this regard, the iterative process of numerical simulations, varying the thermal conductivity until it matches the experimental thermal images, led to the results shown in Figure 7. The graph shows the obtained values of thermal conductivity for all the experimental points, set against the difference in temperature between the upper and lower surfaces of the probe, which is considered a more useful parameter for a clearer and more detailed analysis than the power of the laser. 

The graph shows a significant dispersion of the results for low values on the horizontal axis. This can be explained by the higher relative weight of the uncertainties derived from the experimental measurements. When the temperature difference is small, either due to low laser power or low GO absorption, the variability caused by the resolution of the cameras and the extraction of the profile of temperatures represents a higher percentage of measurement. This effect is reduced when moving to the right side of the graph. For differences in temperature greater than 5 °C, the results of the thermal conductivity converge. To obtain a single representative value, the following fitted curve was used:

(1) $$K=\alpha -\left(\frac{\beta ∆T}{1+\gamma ∆T}\right)$$

where *K* is the thermal conductivity and ∆T is the difference in average temperature between the upper and lower surfaces. The fitting process led to the following constant parameters: α = 0.121, *β* = 0.078, *γ* = 1.316. 

Using this method, the asymptotic value of the thermal conductivity, based on the fitted curve, is 0.064 W/m·K. It is noted that this result is in accordance with both the thermal conductivity, obtained in similar 3D PLA scaffolds generated for tissue engineering applications [28], and the thermal conductivity of additively manufactured PLA probes, the polymer fill ratio of which is around 30% [29].

### 3.2. Effect of the GO Mass on the Photothermal Values

Development of the biocompatible material required the determination of the heating capacity of the PLA + GO probe. It is known that not all the radiation emitted by the laser is transformed into heat and also that the heating capacity is mainly attributable to the GO. The effect of the mass of GO on the thermal behavior of the probe was evaluated, using both experimental and numerical tests, for the set of probes with different mass values of GO, described as SET1 in Table 3 (probe thickness = 2.85 mm).

**Figure 8:**
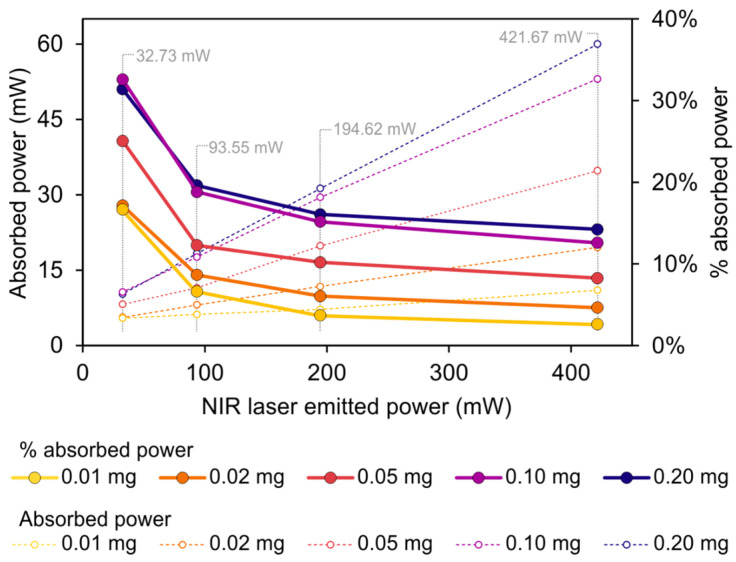
Graph of the total absorbed power (left axis) and the percentage of absorbed power with respect to the total emitted power (right axis) vs the NIR laser emitted power (horizontal axis).

In this regard, the power that the probe absorbs from the laser source is directly related to the temperature profile of the upper surface. Given that the heat flux on this upper surface could not be evaluated easily from the experimental measurements since it depends on the global thermal balance of the entire probe, this value was obtained using numerical simulations. Thus, the integral of the total heat flux on the upper surface of the probe can be reported, which represents the power that is necessary to reproduce the profiles of temperature extracted from the thermal images of the experimental tests. The results are shown in Figure 8.

The dotted lines on the graph show that the total absorbed power increases linearly with the laser-emitted power for all the probes. Nevertheless, this increase is different depending on the mass of GO deposited on the probe. It can be seen that a smaller mass of GO leads to a lesser absorbed power, and also to a less pronounced slope. Conversely, the probes with a larger mass of GO reach higher values of absorbed power.

The solid lines represent the percentage of absorbed power with respect to the total emitted power, that is, a measure of the photothermal conversion efficiency of the GO for this specific application (considering the laser radiation and the convection effect on the upper surface). The value of this efficiency ranges from 2.6% to 14.2% for a high level of emitted power (421.67 mW), and from 16.7% to 32.6% for a low level of emitted power (32.73 mW), indicating that a low level of emitted power leads to higher photothermal conversion efficiency.

Furthermore, it is noted that the percentage of absorbed power increases with the mass of GO; the highest values correspond to the probes with 0.10 mg and 0.20 mg of GO. This analysis enables the determination of the photothermal conversion behavior of the probe, highlighting the fact that the relationship between the increase in the conversion efficiency of GO and the increase in the mass of GO is not linear. In fact, the curves for 0.10 and 0.20 mg are very close. This might demonstrate that a surface concentration of GO in the range of (1.024–2.048) × 10^−3^ mg/mm^2^ would match the optimal absorption capacity for the evaluated range of power, while more GO on the probe would not increase this efficiency.

**Figure 9:**
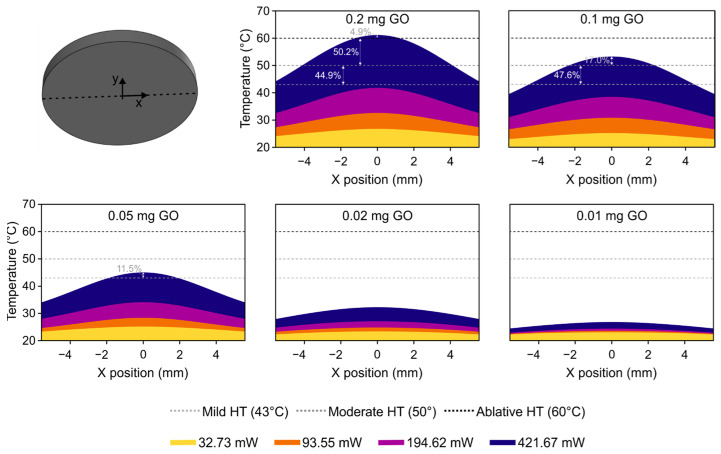
Graph of the profile of the temperature at the bottom surface of the probe for the different values of the mass of GO and laser power. The percentage values represent the percentage of the area of the surface that is above each level of hyperthermia (dotted horizontal lines).

To extend the results obtained from these simulations, an analysis of the temperatures on the bottom surface was performed, since this is the specific factor that determines the degree of hyperthermia that will be reached on the targeted tissue. The graphs in Figure 9 show the profile of temperature on the bottom surface of the probe, representing the percentage of the area of this surface that is above each level of hyperthermia (mild, moderate, and ablative) for the different masses of GO and the various emitted power levels.

The graphs for 0.01 and 0.02 mg of GO indicate that no hyperthermia is reached for any of the tested laser powers. In the case of the probe with 0.05 mg of GO, a mild level of hyperthermia is obtained when the power is at the maximum, but only in a small region (11.5%) in the center of the surface. For 0.1 mg of GO and 421.64 mW of power, the temperature in the central region is above the level of moderate hyperthermia, and 47.6% of the entire surface is under conditions of mild hyperthermia. Finally, considering the maximum power conditions, the probe with 0.2 mg of GO reaches ablative hyperthermia at the center point, a moderate level in more than 50% of the total surface, and mild hyperthermia over the rest of the surface. 

An overall analysis of the thermal behavior of the probes with different amounts of GO shows that not even a mild temperature is reached in any of the probes for an emitted power of 194.62 mW or less.

### 3.3. The Effect of Thickness on Photothermia

**Figure 10:**
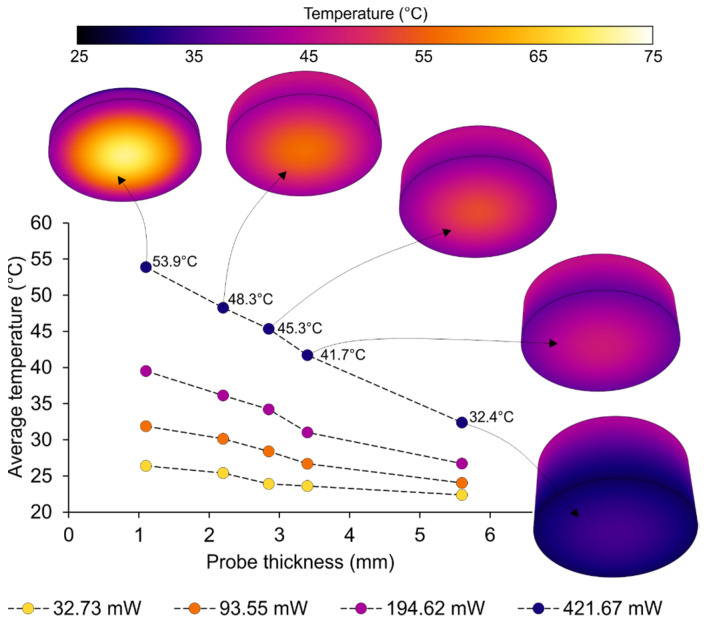
Graph of the average temperature on the bottom surface of the probe, depending on the probe thickness and the emitted laser power.

Apart from the deposited mass of GO, the thickness of the probe is a determinant parameter in terms of the level of hyperthermia reached. This analysis is shown in Figure 10, in which the temperature of the bottom surface is depicted for the different values of probe thickness and emitted power (GO mass = 0.10 mg).

The graph shows the expected decrease in temperature with the increase in probe thickness. Because of the thermal conduction across the material and the larger lateral surface area that is subjected to natural convection, the thickness of the probe is inversely proportional to the average temperature of the bottom surface of the probe. The reduction in the bottom surface temperature is more pronounced for those examples exposed to the higher laser power, where the average temperature of the surface ranges from 53.9 °C for a probe thickness of 1.10 mm, to 32.4 °C for a probe thickness of 5.60 mm. 

**Figure 11:**
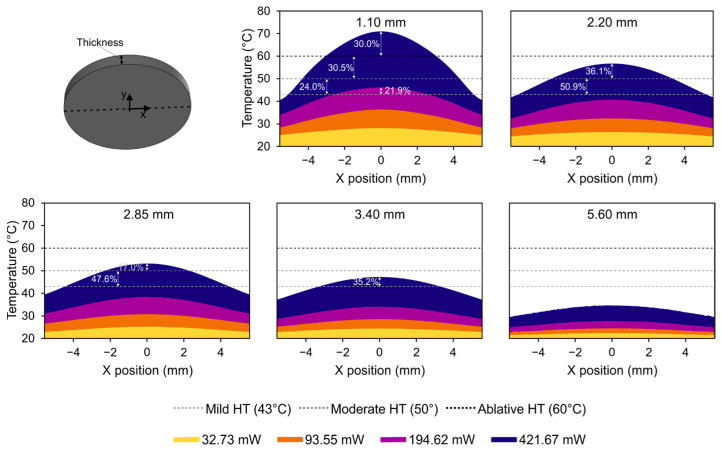
Graph of the profile for temperature on the bottom surface of the probe for the different values of probe thickness and laser power. Percentage values represent the percentage of the area of the surface that was above each level of hyperthermia (dotted horizontal lines).

To evaluate the level of hyperthermia reached on the surface that will be in contact with the affected tissue, the graphs in Figure 11 show the profile of the temperature readings on the bottom surface of the probe, also representing the area of this surface that is above each level of hyperthermia (mild, moderate, and ablative) for the different probe thicknesses and the emitted powers. 

The contours of a probe of 5.60 mm in thickness indicate that the hyperthermia was not reached at any of the emitted powers under test, and, in the 3.40 mm scenario, mild hyperthermia was only reached in the central region at the maximum power level. For the intermediate case with a 2.85 mm probe, the temperature surpasses 50 °C (moderate hyperthermia) in the probe center, while more than 50% of the area is above 43 °C (mild hyperthermia). A similar pattern with higher values is obtained for a probe thickness of 2.20 mm, showing more than 85% of the bottom surface at a temperature above mild hyperthermia. Nonetheless, all the effects mentioned at this point were only observed for the maximum setting of the tested laser power.

Conversely, for a probe thickness of 1.10 mm, mild hyperthermia is reached at the center of the bottom surface for a power of 194.62 mW. This effect favors a reduction in the emitted power by the laser source in terms of achieving the specific target of mild hyperthermia. In this case, when using the maximum power, almost the entire surface is above 43 °C, more than 50% of the area is undergoing moderate hyperthermia, and even 30% of the area in the center region reaches ablative hyperthermia, at above 60 °C. This analysis reveals that, depending on the size of the affected area, an appropriate combination of the mass of the GO and probe thickness must be selected to ensure an appropriate state of hyperthermia in the affected tissue and to establish an optimal level of emitted power from the laser source.

In terms of a comparison with the literature, Li et al. [30] also proposed photothermal therapy to prevent bacterial infections under the mediation of NIR, although in this case, they were using photothermal microspheres, based on the combination of a mixture of black phosphorus nanosheets as photothermal agents and polylactic-glycolic acid. More similarly to the present work, the combination of GO as a photothermal agent and PLA was previously reported by Jin et al. [31]. In this case, photothermal therapy was applied to completely ablate a tumor. They proposed the development of theranostic microcapsules by introducing gold nanoparticles into PLA microcapsules, followed by the deposition of GO onto the microcapsule surface, as performed in the current work. Nevertheless, the present study proves the possibility of not only inducing ablative hyperthermia in a concentrated area and controlling moderate and mild levels of hyperthermia for antibacterial purposes, but also highlights the novelty of the application of nanoparticles through a 3D-printed PLA device.

## 4. Conclusions

The results obtained in the experimental and numerical analyses carried out in the present work prove the use of GO, coated on 3D-printed PLA, as a valid method for NIR photothermal therapy applications. The tests concluded that different levels of hyperthermia will be achieved at the bottom/back face of the GO-PLA discs, considering discs with a diameter of 11.15 ± 0.05 mm in conjunction with an emitted 785 nm NIR laser power ≥ 194.62 mW, a mass of GO ≥ 0.05 mg, and a PLA thickness of ≤3.40 mm. Moreover, the numerical analysis revealed an optimal mass of GO, limited to 1.024–2.048 × 10^−3^ mg/mm^2^; it was proven that a higher content in GO does not increase the conversion efficiency (up to 32.6% for the tested conditions). Based on the experimental tests, a thermal conductivity of 0.064 W/mK for the combination of GO and 3D-printed PLA was also estimated. This value defines the steady-state thermal behavior of the composed material and allows for the simulation of other geometries and conditions that might assist in the design process of new medical devices. Finally, a detailed map of the distribution of temperature across the probes was obtained from the numerical analysis. This way, for example, it was proven that the proposed 785 nm NIR photothermal strategy could achieve the required moderate (50 °C) hyperthermia in a significant area (>50%) of the PLA bottom surface. Therefore, this research opens up new possibilities for photothermal therapy application concerning the local treatment of infected tissues, especially in the oral cavity, taking advantage of the versatility of 3D printing to design devices that are adapted to each patient.

## Figures and Tables

**Table 1:** Technical data of the natural polylactic acid (PLA) Luminy^®^ LX175 pellets, taken from the product data-sheet. https://www.totalenergies-corbion.com/media/eushodia/pds-luminy-l175-190507.pdf (accessed on 15 January 2023).

PLA Luminy^®^ LX175	Value
Material density	1.24 g/cm^3^
Residual monomer	≤0.3%
Tensile strength	50 MPa
Tensile modulus	3500 MPa
Melting temperature	175 °C
Glass transition temperature	60 °C

**Table 2:** Main 3D printer parameters used.

Tumaker Voladora NX Pellet	Parameter Values
Nozzle diameter	0.80 mm
Nozzle temperature	190–230 °C
Bed temperature	45 °C
Infill pattern	Rectilinear (angle of 45/−45°)
Speed	60 mm/s
Layer height	0.20 mm

**Table 3:** Summary of the two sets of GO-PLA probes.

	Probe Thickness (mm)					GO Mass (mg)				
SET1	2.85					0.01	0.02	0.05	0.10	0.20
SET2	1.10	2.20	2.85	3.40	5.60	0.10				

## Data Availability

Not applicable.
